# Configuration Optimisation of Laser Tracker Location on Verification Process

**DOI:** 10.3390/ma13020331

**Published:** 2020-01-10

**Authors:** Sergio Aguado, Pablo Pérez, José Antonio Albajez, Jorge Santolaria, Jesús Velázquez

**Affiliations:** Design and Manufacturing Engineering Department, Universidad de Zaragoza, María Luna 3, 50018 Zaragoza, Spain; pperezm@unizar.es (P.P.); jalbajez@unizar.es (J.A.A.); jsmazo@unizar.es (J.S.); jesusve@unizar.es (J.V.)

**Keywords:** laser tracker, machine tool, uncertainty, Monte Carlo method, verification

## Abstract

Machine tools are verified and compensated periodically to improve accuracy. The main aim of machine tool verification is to reduce the influence of quasi-static errors, especially geometric errors. As these errors show systematic behavior, their influence can be compensated. However, verification itself is influenced by random uncertainty sources that are usually not considered but affect the results. Within these uncertainty sources, laser tracker measurement noise is a random error that should not be ignored and can be reduced through adequate location of the equipment. This paper presents an algorithm able to analyse the influence of laser tracker location based on nonlinear optimisation, taking into consideration its specifications and machine tool characteristics. The developed algorithm uses the Monte Carlo method to provide a zone around the machine tool where the measurement system should be located in order to improve verification results. To achieve this aim, different parameters were defined, such as the number of tests carried out, and the number and distribution of points, and their influence on the error due to the laser tracker location analysed.

## 1. Introduction

Machine tools (MTs) are increasingly implemented in the industrial sector, which is itself increasingly competitive and seeks to increase production at a lower cost. For this, detection and reduction of MT errors is necessary.

Currently, there are two different ways to obtain MT geometric errors: direct and indirect measurement methods. Direct measurement methods consist of measuring the influence of every individual error from each axis in a particular position of the workspace of the MT [1]. Alternatively, indirect measurement methods obtain the joint influence of MT geometric errors based on multi-axis movement and its kinematic model. These are more widely used, especially in long range MTs, where direct methods require large scales, expensive dimensional measurement systems, and more time to check them [1]; so, the limitations of direct measurement cause indirect measurement to prevail in this type of machine.

Volumetric verification, using a laser tracker (LT) as a measurement system, is based on indirect measurement of geometric errors, characterising their combined effect [2]. So, the accuracy of verification results depends, among others, on errors of the MTs but also on the errors of the measurement system used. These latter errors are often ignored, and it is assumed that the performance of the measurement system is sufficiently accurate.

All measurements have a degree of uncertainty made up of systematic and random error sources. The systematic errors of LTs, such as environmental conditions or component assembly, can be estimated and compensated by software. However, random errors, such as LT measurement noise, cannot be compensated but can be reduced by the appropriate location of the measurement system, so improving verification results [3].

To find the optimal LT location, the technical specifications of the encoders, the characteristics of the MTs [4], physical restrictions such as the range of the laser tracking receiver [5], and even temperature variations [6] are required.

This paper presents a developed algorithm able to determine the influence of LT measurement noise on the verification results. The algorithm takes into consideration LT characteristics and MT workspace. In addition, the developed software uses the Monte Carlo method to provide the area where the LT should be located with its probability distribution function (PDF).

## 2. Materials and Methods

### 2.1. LT as Verification Measurement System

**Figure 1:**
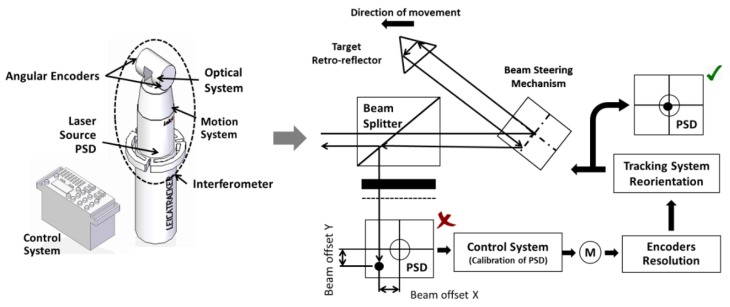
Errors due to encoders and sensor. Position-sensitive device (PSD).

A LT is a portable measurement system that provides, in a spherical coordinate system, the position of a measured point. It is often composed of a laser mechanism oriented by means of angular encoders, an interferometer block, a position-sensitive device (PSD), optics responsible for the beam division, a reflector, and a control unit. Point coordinates are determined by comparing the measurement beam with the reference beam from the laser interferometer together with the combination of the azimuth and polar angle encoders of its head, which provide two rotational degrees of freedom of the LT (Figure 1).

#### 2.1.1. Error Sources in an LT

Like any other measurement system, LTs are affected by systematic and random errors. Currently there are three standards concerning performance evaluation of LTs: ASME B.89.4.19-2005 [7], VDI/VDE 2617-10 [8], and ISO 10360-10 [9]. These three standards provide different tests to verify the performance of an LT according to the specifications of the manufacturer, reducing the influence of LT errors on the measurement.

Gallagher [10] classified error sources as: angular encoder, tracking system, and component misalignments. Knapp [11] divided sources of errors into those due to environmental factors, data capture, approximations, and simplifications.

Errors due to the interferometer and optics are the result of environmental influences and LT calibration. Atmospheric effects, variations in the speed of light, and turbulence affect the physical characteristics of the laser beam [12]. The environmental conditions, pressure, temperature, and humidity produce variations in the refractive index of the air. These variations result in errors in the laser wavelength and finally, in the measured distance [10]. In a factory workshop without a temperature-controlled environment, the temperature can significantly fluctuate through the day. In [13] authors reported an example of an aircraft assembly facility with temperature variations of 8 °C over 4 h and variation on the vertical directions of 2.2 degrees. During the aircraft assembly process, if the beginning and the ending temperatures of the measurement survey vary by more than 2.2°, then the survey is considered void and has to be repeated. Nevertheless, environmental conditions present a systematic behaviour described analytically. Therefore, the LT control unit can compensate for this influence due to its meteorological position.

Moreover, installation of LT optics introduces a series of intrinsic errors such as the Abbe error, cosine error, and depth error. If the reflector does not move parallel to the measurement axis, a cosine error will occur. In the same way, if the reflector does not move along the measurement axis of the interferometer, an Abbe error occurs. Similarly, an error of calibration between the home and reflector provides a depth error that will be transferred to all measurement points.

Additionally, the main sources of error in a PSD are its resolution and the calibration procedure that was used to determine the relationship between the sensor output and the beam offset from the centre of the target used to calculate the measured point. This is minimised by the sub-system, consisting of two stepper motors, two optical angular encoders, and a motion control card. The two motors produce the azimuthal and polar rotation of the beam tracking system, allowing the laser beam to move towards the centre of the PSD target, minimising the offset. Depending on the resolution of the encoders used, a better adjustment of the offset will be made (Figure 1).

#### 2.1.2. LT Location in the Verification Process

The presence of LTs is increasing daily in machining and metrology companies, as tools to improve the accuracy of MTs through verification. Although LTs can be used to measure errors through geometric or pseudo-geometric verification [14], they are more frequently used in volumetric verification.

For this, the equipment should be located inside the MT kinematic chain in the same place as the workpiece [2,15]. MT kinematic chains are classified based on the movement of the workpiece and tool. The MT presented in this paper has an XFYZ configuration, where F determines the fixed part of the machine, X represents the axis that moves the part and the LT during the verification process, and Y and Z represent the axes that move with the tool [2].

The MT+LT kinematic chain mathematically links the tool centre point with the part of the machine, taking into consideration the sequence of movement and geometric errors of the MT (Equation (1)):

(1) $$\bar{X}+\bar{R_{x}}\;\bar{T_{lt}}+\bar{R_{x}}\;\bar{R_{lt}}\;\bar{X_{lt}}\;=\;\bar{Y}+\bar{R_{y}}\;\bar{Z}\;+\;\bar{R_{y}}\;\bar{R_{z}}\;\bar{T}$$

where $\bar{X},\bar{Y},$ and $\bar{Z}$ represent the translational vectors of the X, Y, and Z axes, respectively, with their geometrical errors and nominal displacements. $\bar{R_{x}},\;\bar{R_{y},}$ and $\bar{R_{z}}$ are the rotational matrices of the X, Y, and Z-axes defined by their rotational errors. $\bar{X_{lt}}$ and $\bar{T_{lt}}$ represent the translation and rotational matrices between the LT and the origin of the MT coordinate system. Finally, $\bar{T}$ describes the offset of the tool [2].

**Figure 2:**
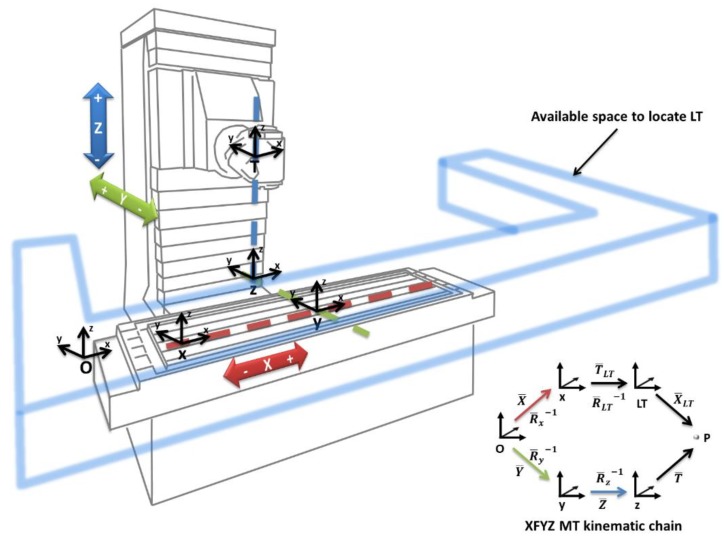
Machine tools (MTs) kinematic chain with XFYZ configuration, where F determines the fixed part of the machine, X represents the axis that moves the part and the LT during the verification process, and Y and Z represent the axes that move with the tool. Laser tracker (LT).

Figure 2 shows the physical space available to locate the LT. Additionally, LT angular limitations such as maximum and minimum azimuth and polar encoders, and minimum radial distance or height couplings should be taken into account when locating the LT.

#### 2.1.3. Influence of LT Location on MT Volumetric Verification

While systematic errors can be compensated by the LT control unit, other errors, such as the angle of incidence on the retro-reflector, or errors in the PSD sensor, due to angular encoders and the interferometer, produce a non-systematic error commonly known as measurement noise.

The influence of measurement noise on measured points is modelled with Equations (2)–(4). These Equations link data from the encoders and radial distances with their uncertainty, providing the uncertainty of a measured point in Cartesian coordinates:

(2) $$u_{x}^{2}=u_{r}^{2}\cdot \sin^{2}\theta \cdot \cos^{2}\varphi +u_{\theta }^{2}\cdot r^{2}\cdot \cos^{2}\theta \cdot \cos^{2}\varphi +\;u_{\varphi }^{2}\cdot r^{2}\cdot \sin^{2}\theta \cdot \sin^{2}\varphi$$

(3) $$u_{y}^{2}=\;u_{r}^{2}\cdot \sin^{2}\theta \cdot \sin^{2}\varphi +u_{\theta }^{2}\cdot r^{2}\cdot \cos^{2}\theta \cdot \sin^{2}\varphi +\;u_{\varphi }^{2}\cdot r^{2}\cdot \sin^{2}\theta \cdot \cos^{2}\varphi$$

(4) $$u_{z}^{2}=\;u_{r}^{2}\cdot \cos^{2}\theta +\;u_{\theta }^{2}\cdot r^{2}\cdot \sin^{2}\theta$$

where *r* is the radial measured distance, $u_{r}$, the radial uncertainty, θ, the azimuth angle, $u_{\theta }$, the azimuth angle uncertainty, φ, the polar angle, and $u_{\varphi }$, the polar angle uncertainty.

As LTs work with an absolute coordinate system and MT to verify, nominal MT points are not in the same coordinate system when are measured. So, their real uncertainty depends on verification of the LT location in the MT workspace.

### 2.2. LT Location Algorithm

#### 2.2.1. Working Principles

The main aim of the developed algorithm was to provide the location of the area where the influence of the measurement system noise is smaller than the admissible error. The Guide to the Expression of the Uncertainty in Measurement (GUM) provides a framework for evaluating and expressing measurement uncertainty evaluating type A, type B, and combined uncertainties. Type A uncertainty is evaluated using statistical means, while type B is only evaluated based on experience or other information. However, the estimation of uncertainties using GUM relies on assumptions, such as non-linearity of the mathematical model, that are not always fulfilled [16]. In these cases, supplement 1 to the GUM describes the problem of uncertainty evaluation in terms of probability density functions to obtain the best estimate thorough the Monte Carlo method.

In this case, the influence of measurement noise is obtained through optimisation based on the Levenberg–Marquardt method, taking into consideration the following information:

1. Nominal MT verification points.
2. LT characteristics and limitations.
3. Limits of LT location.
4. Optimisation criteria to minimise the influence of uncertainty.
5. Number of Monte Carlo tests used to determine the location area.

**Figure 3:**
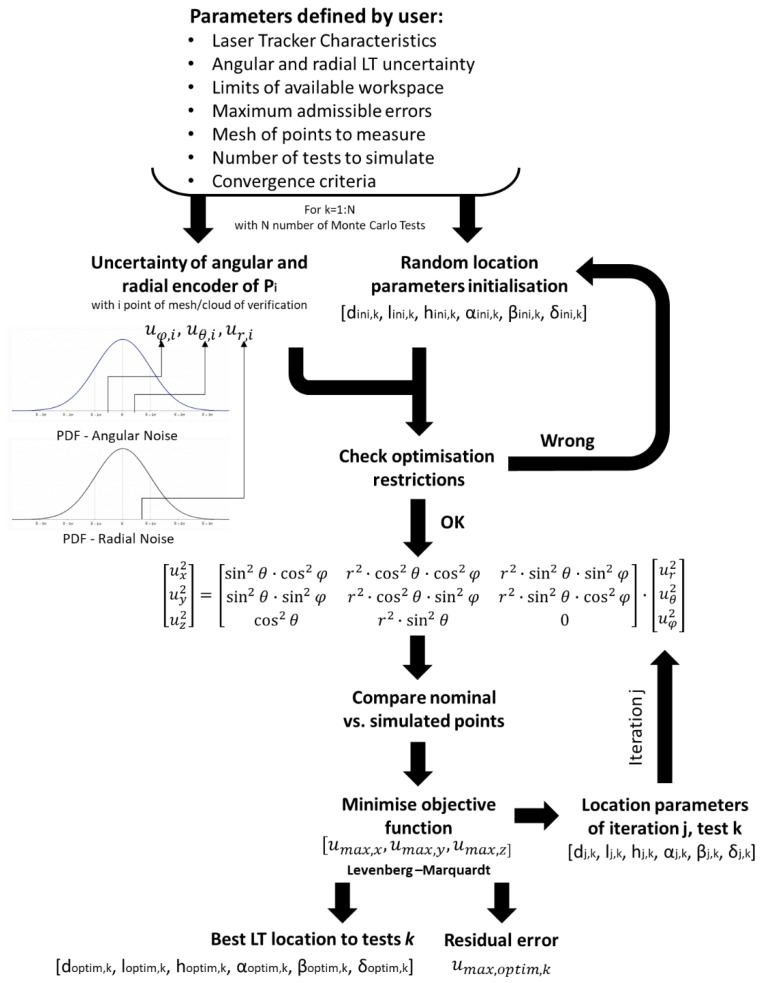
Working principle of the location algorithm.

The working principle of the developed algorithm is presented in Figure 3. First, the user introduces configuration parameters: LT characteristics, angular and radial uncertainties, limits of available workspace, maximum admissible error, mesh of measurement points, number of tests to simulate, and convergence criteria.

Then, the algorithm begins to perform a test loop for k = 1 to k = n, with n being the number of tests defined by the user. Next, the algorithm randomly takes a value from the angular and radial PDF for each point. These values will be fixed throughout the test k, changing from one test to the next. Simultaneously, the algorithm looks for a random position within the available space for the initial location parameters. These parameters are defined by a 1 × 6 vector (d, l, h, α, β, δ), which transforms coordinates from the MT coordinate system to the LT coordinate system. Parameters d, l, and h represent a translation between the MT coordinate system and the LT coordinate system on the x, y, and z-axes, respectively, and α, β, and δ are the Euler angles that relate the orientation of the LT coordinate system to that of the machine tool, rotating first around the x-axis, then the y-axis, and finally around the z-axis.

If the restrictions are met, then the uncertainty of each point is calculated using Equations (2)–(4). If not, the algorithm looks for others. Afterward, the objective function (5) is calculated:

(5) umax=(umax,x2+umax,y2+umax,z2)12.

This function is defined from a 1 × 3 vector made up from ($u_{max,x},u_{max,y},\;u_{max,z}$), considering the most restrictive criteria as admissible errors (all maximum uncertainties are at the same point). In this way, the influence of measurement uncertainty in the verification points will always be equal to or less than the residual optimisation result.

During optimisation, the algorithm modifies in each iteration *j*, the location parameters d, l, h, α, β, and δ, changing the spherical coordinates r, θ, and φ of each point to minimise the uncertainty influence.

When the optimisation is finished, the algorithm returns the optimisation parameters with the residual error. If the residual error is less than the admissible error introduced by the user, the algorithm stops. If not, the software divides the MT workspace into two areas and repeats the process. Moreover, the algorithm provides the PDF that defines the uncertainty behaviour depending on the location of the LT.

#### 2.2.2. Case Study

**Figure 4:**
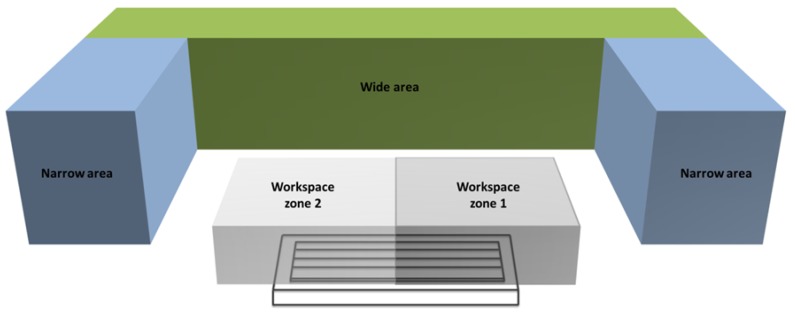
Admissible LT locations areas and workspace zones.

All tests carried out had common simulation conditions: (a) the workspace to verify, defined by its limits of movement: 0 mm ≤ x ≤ 1500 mm, 0 mm ≤ y ≤ 600 mm, and 0 mm ≤ z ≤ 400 mm. (b) The available workspace around the MT where the LT could be located. This space was divided into two areas: narrow and wide. The narrow area had as available location parameters: 350 mm ≤ h ≤ 2000 mm, −500 mm ≤ d ≤ 2000 mm, and −2000 mm ≤ l ≤ −500 mm. The wide area had as available location parameters: 350 mm ≤ h ≤ 2000 mm, −2000 mm ≤ d ≤ −500 mm, and −500 mm ≤ l ≤ 2000 mm. As an additional restriction, the algorithm did not allow location of the LT inside the verification workspace (Figure 4). (c) The LT limits introduced in the algorithm were: azimuth angle θ −235° ≤ θ ≤ 235° and polar angle φ −60° ≤ φ ≤ 77°. (d) The PDF that defined the angular and radial uncertainties were normal distributions with *µ* = 20 µrad and *σ* = 1.5 µrad for the angular encoder and 4 µm ± 0.8 µm/m for the radial. Finally, the optimisation criteria limits were the same for all tests. These limits were: maximum iterations set at 1000, the minimum parameter variation set as 1 × 10^−12^ and the minimum objective function variation set as 1 × 10^−5^.

**Figure 5:**
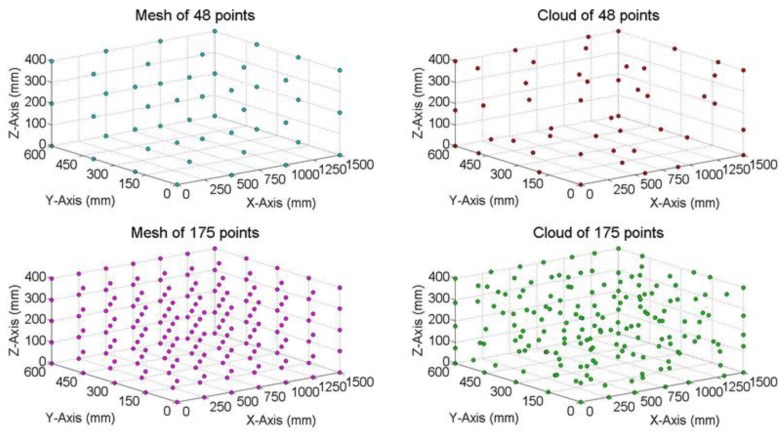
Distribution and number of points studied.

This paper studied the influence of the spatial distribution of MT workspace points, the number of points used to determine the LT location and the number of Monte Carlo tests used. Point distributions can be a mesh or a cloud. The number of points studied were 48 or 175 (Figure 5), and the number of Monte Carlo tests carried out were 100, 1000, and 10,000.

## 3. Results

### 3.1. Uncertainty Due to LT Location

The first tests carried out to study the uncertainty of locating an LT in the narrow and wide areas used a mesh distribution of points, with 175 points and 10,000 Monte Carlo tests to obtain optimal values of d, l, h, α, β, and δ.

**Figure 6:**
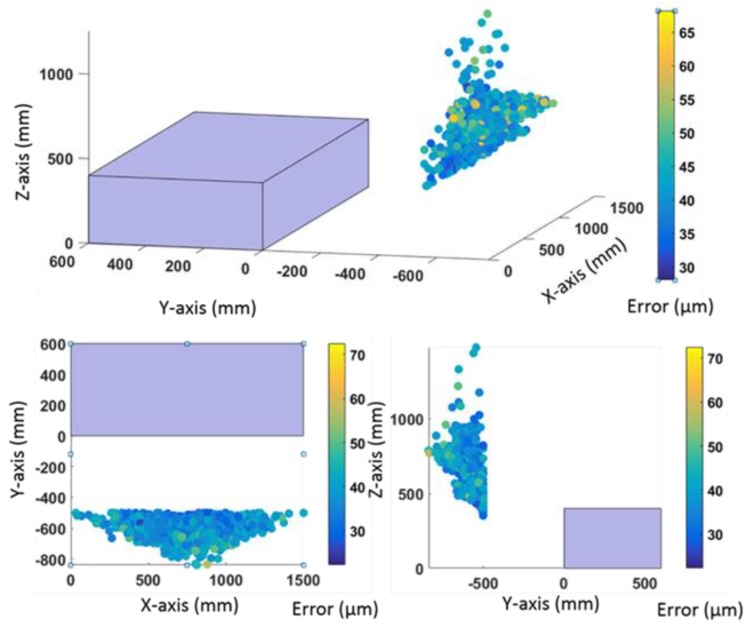
Error and LT location area in the wide zone using a laser–residual error.

**Figure 7:**
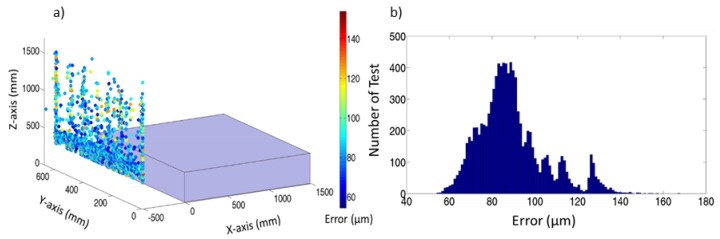
(**a**) Error and LT location area in the narrow zone using an LT. (**b**) Histogram of residual error.

As the colourmap of Figure 6 shows, when the LT is located in the wide area the error range was from 27.1 to 72.0 μm. That is to say, the test with the least influence of LT noise with specific values of *u*_*r*__,__*i*_, *u*_*θ*__,__*i*_, and *u*_*φ*__,__*i*_ with *i* = 1.175, provides a maximum uncertainty value of 27.1 μm, while the optimal parameters d, l, h, α, β, and δ in the test with the maximum uncertainty produce a value of 72.0 μm, taking into account that each test had different initial location parameters in the available workspace of Figure 4, with an initial error higher than final one presented in Figure 6 and Figure 7 (residual error).

When the LT is located in the wide area (Figure 6) there is a zone of conical shape where the tests present a high concentration of optimal locations with uncertainty values between 27.1 and 72.0 μm. So, the LT should be located in the wide area between −830 mm ≤ d ≤ −500 mm, 500 mm ≤ l ≤ 1000 mm, and 700 mm ≤ h ≤ 850 mm where the cone is registered.

When the LT is located in the narrow area, as shown in Figure 7a, noise uncertainty due to LT location increases from 56.2 μm to 170.5 μm. However, when the LT is located in the narrow zone there is an area to locate the LT of rectangular shape with l = −500 mm, 350 mm ≤ h ≤ 600 mm, and 0 mm ≤ d ≤ 600 mm, where the uncertainty is less than 115 μm. Figure 7b shows the histogram of residual errors, which allows study of the PDF that defines the behaviour of LT location influence. These errors are similar in the narrow and wide areas.

When the residual error is higher than the introduced admissible error, the algorithm divides the verification area so that x = 750 mm (named as workspace 1 and workspace 2 in Figure 4). Then, the software analyses the influence of LT on these areas as independent workspaces, maintaining the location conditions. Table 1 compares the maximum and minimum errors when the MT workspace is divided.

This shows that there is a relevant reduction inside the new workspace near to the LT in the narrow zone, where the minimum influence is reduced from 56.2 μm to 20.6 μm and the maximum is from 170.5 μm to 47.1 μm in Workspace 1, a reduction of approximately 70%. In the wide zone, Workspace 2, the reduction is not meaningful, around 3%. If two LTs are located on wide zones, the influence of LT uncertainty, minimum and maximum, is around 20% and 25% smaller, respectively.

### 3.2. Influence of Design Conditions on Location Results

Tests carried out using a unique LT in narrow or wide areas, as presented in the previous section, required a computational cost of around 6 h using a commercial PC. This was increased when the MT workspace was divided.

This value is too high for in situ machine verification. To reduce it, several configurations were tested, to study the influence of:

- Number of tests (100, 1000, or 10,000).
- Number of points (48 or 175).
- Distribution of points (mesh or cloud).
- Available workspace (narrow or wide).
- Number of LTs (1 or 2).

Due to the very large number of tests carried out, only the more relevant ones are presented here. Table 2 presents the computational cost of different design configurations depending on the number of points and tests. The distribution of points did not have a significant influence on computational cost.

**Figure 8:**
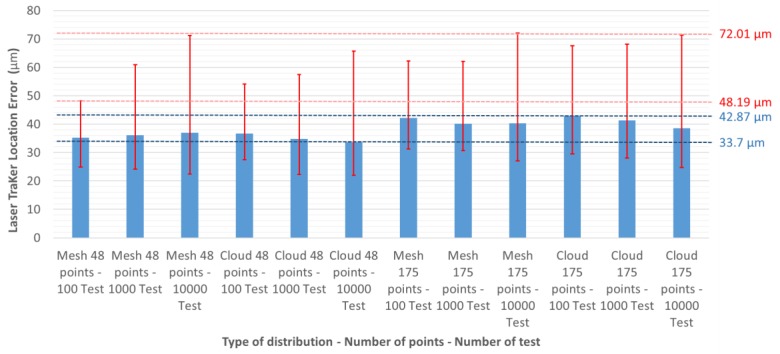
Error of LT location in the wide zone using one LT, depending on the type and number of tests, and the distribution and number of points.

To study whether errors are significantly affected by different design configurations is necessary. Figure 8 shows the errors introduced by a unique LT in the wide zone, depending on the test configuration. The blue column represents the mean error of tests performed for each configuration. The red vertical lines show the range of the error produced for each configuration. The upper end is the maximum error chosen for a test and the lower end the minimum. For example, the design of a configuration consisting of a mesh of 175 points and 10,000 tests has an average error of 40.2 µm, a maximum error of 72.0 µm, and a minimum error of 27.1 µm, with a total range of 44.9 µm. The ninth column of the graph in Figure 8 is equivalent to Figure 6 and the results of the lower left configuration in Table 1.

In Figure 8 it can be seen that the mean error due to LT location is similar in all configurations, with a range of 33.7–42.87 µm. However, the maximum error range is 48.19–72.01 µm. Also, there seems to be a lower limit around the zone of 20 µm.

The configuration parameters that have more influence on measurement error due to LT location were studied based on statistical design of experiment (DOE) [17]. Three input parameters were studied: parameter *A* = points distribution: mesh or cloud; parameter *B =* number of points: 48 or 175; and parameter *C* = number of tests: 100 or 10,000.

**Figure 9:**
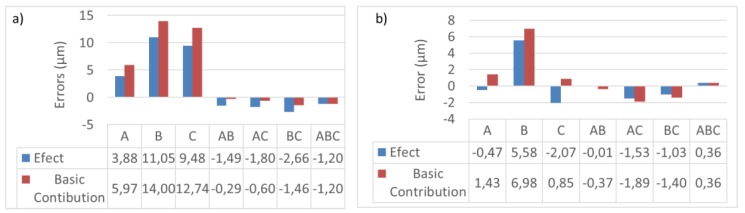
Effect and basic contribution of point distribution, number of points and number of tests on maximum error (**a**) and average error (**b**).

Figure 9 shows the results of the DOE applied to the maximum and mean error, representing the effect and the basic contribution of each parameter. The most relevant is the number of points in both cases. The greater the number of points, the greater the error. The number of tests has a large influence on maximum error but not on the average, causing different effects. The type of point distribution is the least relevant parameter in error due to LT location. The combination has the opposite effect on maximum error, compared to individual (Figure 9a). Similar behaviour is observed in the averages (Figure 9b).

To study whether the influence of these parameters can be modelled as lineal regressions, the Scheffler regression function was used [18] to give the maximum and average errors (Equations (6) and (7)):

(6) maxerror (µm)=62.77+1.94A+5.225B+4.740C−0.745AB−0.900AC−1.330BC−0.600ABC

(7) average error (µm)=38.187−0.234A+2.789B−1.036C−0.004AB−0.764AC−0.516BC+0.181ABC. 

To validate the adequacy of Equations (6) and (7), tests of different configurations, with 1000 tests, 48 and 175 points, and mesh and cloud distributions were used. In Table 3 we can see that these parameters do not have a linear behaviour, as might be anticipated from Figure 8.

Table 4 shows the influence, in the wide zone, of distribution, number of points, and number of tests if the MT workspace is divided. Conclusions drawn from the results obtained are the same as those of the wide zone using one LT. The same is seen with the DOE tests.

**Figure 10:**
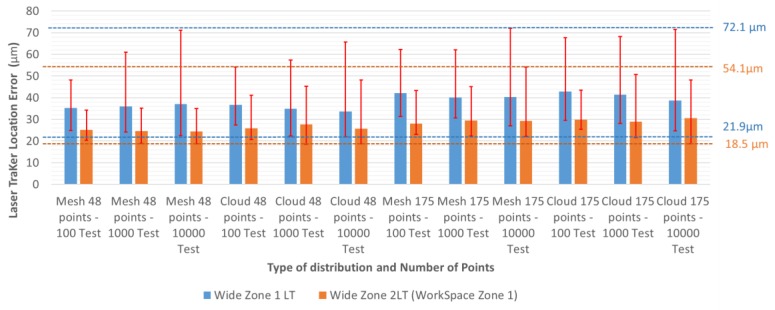
Error of the LT location in the wide zone. One zone vs. two zones with different configurations.

Similar results were obtained when dividing the workspace volume into two areas: regardless of the design, there is a reduction in the maximum and average values of the error introduced. The error in Zone 1, compared to using only one LT, reduced from 34% to 21% in average values and from 50% to 21% in maximum error. In Zone 2, the average error was reduced from 42% to 22% and the maximum error from 50% to 21% (Table 4, Figure 10). As shown in Figure 10, when the MT workspace is divided, the range of error is reduced from 50.2 µm to 35.6 µm, and a minimum error support zone is also found at around 20 µm.

## 4. Discussion

Tests carried out show that there is no unique optimal position to locate the LT. As its uncertainty is defined by a PDF, each verification point will be affected by different values in each test. Therefore, there is one area where the PDF of LT influence is optimum. This depends on the measurement systems characteristic, therefore, the first step is to provide an adequate equipment characterization.

When only one LT is used to verify the whole MT workspace, the verification results can be improved by locating the LT in the wide area, inside the estimated zone with a cone shape. The Monte Carlo analysis provides an uncertainty range from 27.1 to 72 µm, providing a maximum error around 60% smaller than that obtained when the LT is located in the narrow area.

If the residual error obtained is too high, the division of the workspace into two zones provides an improvement in uncertainty due to LT location. This is especially relevant in the narrow zone, where the maximum error in workspace 1 is reduced by around 70%. If two LTs are used and located in the wide zone, their influence compared to the use of just one LT is improved by around 25%. Thus, the use of two workspaces and two LTs reduces their location influence. These results show that, in these cases, the greater the distance of measurement, the greater the mistake is committed. Therefore, the LT might be placed near the workspace to verify. One should recall that there is a minimum distance allowable for each equipment.

Tests carried out to study the influence of design configuration show that the number of points is the most relevant parameter, followed by the number of tests, and the points distribution. Moreover, we demonstrate that their relationship cannot be modelled as linear regression functions. Therefore, users should assess the computation costs against the accuracy of the method to determine the configuration parameters.

## Figures and Tables

**Table 1:** Influence of LT uncertainty depending on location and number of devices.

Zones	Workspace Divided Into Two Zones; Two LTs				Workspace 1 Zone, 1 LT	
	Min. Error (µm)		Max. Error (µm)		Min. Error (µm)	Max. Error (µm)
	Space 1	Space 2	Space 1	Space 2	Unique Workspace	Unique Workspace
Narrow	20.6	54.0	47.1	167.9	56.2	170.5
Wide	22.1	23.7	54.1	49.9	27.1	72.0

**Table 2:** Computational time for different test configurations.

Points	Tests	Time	Points	Tests	Time
48	100	1′35″	175	100	4′20″
48	1000	14′32″	175	1000	42′31″
48	10,000	2 h 3′11″	175	10,000	6 h 37′56″

**Table 3:** Adequacy of Scheffler regression functions for the average and maximum error values.

Configuration			Real Value		Estimated Value	
Type	Points	Tests	Mean (µm)	Max. (µm)	Mean (µm)	Max. (µm)
Mesh	48	1000	36.1	60.9	35.3	49.4
Cloud	48	1000	34.8	57.4	36.4	55.2
Mesh	175	1000	40.2	72.0	42.2	63.1
Cloud	175	1000	41.3	68.2	42.5	67.9

**Table 4:** Influence of design parameters on wide zone LT location area and after division of the MT workspace.

Configuration			Wide Zone-Workspace Zone 1			Wide Zone-Workspace Zone 2		
Type	Points	Tests	Mean (µm)	Max. (µm)	Min. (µm)	Mean (µm)	Max. (µm)	Min. (µm)
Mesh	48	100	25.2	34.3	20.3	25.2	34.3	20.3
Mesh	48	1000	24.6	35.1	18.9	24.6	35.1	18.9
Mesh	48	10,000	24.4	35.1	18.6	21.3	35.1	18.6
Cloud	48	100	25.9	41.2	20.8	24.8	41.2	19.4
Cloud	48	1000	27.6	45.3	18.5	27.1	45.4	18.7
Cloud	48	10,000	25.7	48.1	18.7	25.3	48.2	18.90
Mesh	175	100	28.1	43.3	23.0	31.9	44.3	25.3
Mesh	175	1000	29.5	45.1	22.3	30.4	46.2	23.3
Mesh	175	10,000	29.2	54.1	22.1	29.5	49.8	23.7
Cloud	175	100	29.9	43.4	25.4	29.5	44.6	25.5
Cloud	175	1000	28.9	50.7	21.6	29.7	51.4	22.5
Cloud	175	10,000	30.5	48.1	18.7	30.1	48.2	19.3
